# Reconciling newborn screening and a novel splice variant in *BTD* associated with partial biotinidase deficiency: a BabySeq Project case report

**DOI:** 10.1101/mcs.a002873

**Published:** 2018-08

**Authors:** Jaclyn B. Murry, Kalotina Machini, Ozge Ceyhan-Birsoy, Amy Kritzer, Joel B. Krier, Matthew S. Lebo, Shawn Fayer, Casie A. Genetti, Grace E. VanNoy, Timothy W. Yu, Pankaj B. Agrawal, Richard B. Parad, Ingrid A. Holm, Amy L. McGuire, Robert C. Green, Alan H. Beggs, Heidi L. Rehm, The BabySeq Project Team

**Affiliations:** 1 Laboratory for Molecular Medicine, Cambridge, Massachusetts 02139, USA;; 2 Department of Pathology, Brigham & Women's Hospital, Boston, Massachusetts 02115, USA;; 3 Department of Pathology, Memorial Sloan Kettering Cancer Center, New York, New York 10065, USA;; 4 Division of Genetics and Genomics, Boston Children's Hospital, Massachusetts 02115, USA;; 5 Harvard Medical School, Boston, Massachusetts 02115, USA;; 6 Division of Genetics, Department of Medicine, Brigham and Women's Hospital, Boston, Massachusetts 02115, USA;; 7 Division of Genetics and Genomics, The Manton Center for Orphan Disease Research, Boston Children's Hospital, Boston, Massachusetts 02115, USA;; 8 Department of Neurology, Boston Children's Hospital, Boston, Massachusetts 02115, USA;; 9 Division of Newborn Medicine, Boston Children's Hospital, Boston, Massachusetts 02115, USA;; 10 Department of Pediatric Newborn Medicine, Brigham and Women's Hospital, Boston, Massachusetts 02115, USA;; 11 Center for Medical Ethics and Health Policy, Baylor College of Medicine, Houston, Texas 77030, USA;; 12 The Broad Institute of MIT and Harvard, Cambridge, Massachusetts 02141, USA; 14 Boston Children's Hospital, Boston, Massachusetts 02115, USA; 15 Brigham and Women's Hospital, Boston, Massachusetts 02115, USA; 16 Laboratory for Molecular Medicine, Cambridge, Massachusetts 02139, USA; 17 Oregon Health and Science University, Oregon 97239, USA; 18 Baylor College of Medicine, Houston, Texas 77030, USA; 19 Broad Institute, Massachusetts 02141, USA; 20 Massachusetts General Hospital, Boston, Massachusetts 02115, USA

**Keywords:** abnormal enzyme/coenzyme activity

## Abstract

Here, we report a newborn female infant from the well-baby cohort of the BabySeq Project who was identified with compound heterozygous *BTD* gene variants. The two identified variants included a well-established pathogenic variant (c.1612C>T, p.Arg538Cys) that causes profound biotinidase deficiency (BTD) in homozygosity. In addition, a novel splice variant (c.44+1G>A, p.?) was identified in the invariant splice donor region of intron 1, potentially predictive of loss of function. The novel variant was predicted to impact splicing of exon 1; however, given the absence of any reported pathogenic variants in exon 1 and the presence of alternative splicing with exon 1 absent in most tissues in the GTEx database, we assigned an initial classification of uncertain significance. Follow-up medical record review of state-mandated newborn screen (NBS) results revealed an initial out-of-range biotinidase activity level. Levels from a repeat NBS sample barely passed cutoff into the normal range. To determine whether the infant was biotinidase-deficient, subsequent diagnostic enzyme activity testing was performed, confirming partial BTD, and resulted in a change of management for this patient. This led to reclassification of the novel splice variant based on these results. In conclusion, combining the genetic and NBS results together prompted clinical follow-up that confirmed partial BTD and informed this novel splice site's reclassification, emphasizing the importance of combining iterative genetic and phenotypic evaluations.

## CASE PRESENTATION

Here we describe a newborn female who was delivered via vaginal delivery at 40 wks 5 d gestational age to a 29-yr-old mother after an uncomplicated pregnancy. The physical exam at birth was unremarkable, and all growth parameters were proportionate and appropriate for her gestational age. She was enrolled in the well-baby cohort of the BabySeq Project and randomized to undergo whole-exome sequencing with analysis limited to genes strongly associated with pediatric-onset disorders. There were no major health concerns, surgeries, or hospitalizations between her well-baby nursery discharge and the time genomic results were reported at 2.5 mo of age. Both maternal and paternal families were of European ancestry, with no known consanguinity. Family history was significant for a 17-mo-old brother with severe eczema and two distant paternal relatives reported to have alopecia (one of whom had onset as a teenager).

## TECHNICAL ANALYSIS AND METHODS

In the healthy proband, both *BTD* germline variants (p.Arg538Cys and c.44+1G>A) were identified through exome sequencing as part of the BabySeq Project. Testing was performed as described previously (Ceyhan-Birsoy et al. 2017). Variants were validated by Sanger sequencing using DNA extracted from peripheral blood-derived genomic DNA. Sanger sequencing was also performed on saliva-derived genomic DNA from parents to determine phasing for both variants. Mandated newborn screening (NBS) was conducted at The New England Newborn Screening Laboratory for the Massachusetts Newborn Screening Program (https://nensp.umassmed.edu), and biotinidase deficiency (BTD) was screened by an enzymatic activity assay conducted on samples extracted from dried blood spots.

## VARIANT INTERPRETATION

Two heterozygous variants in the *BTD* gene encoding the biotinidase enzyme were identified in the subject. One was a known pathogenic missense variant (NM_000060.4:c.1612C>T, p.Arg538Cys) (ClinVar Variation ID: 1898; https://www.ncbi.nlm.nih.gov/clinvar) that has been previously reported in at least five compound heterozygous and two homozygous individuals with severe BTD. It is the second most common pathogenic variant in the *BTD* gene and is associated with profound BTD. Pomponio et al. (1997) concluded that individuals with this variant are reported to have undetectable biotinyl transferase activity in their serum and <10% of mean normal biotinyl-hydrolase activity. This variant is identified in the heterozygous state on 14/121,552 non-Finnish European chromosomes in the Genome Aggregation Database (gnomAD, http://gnomad.broadinstitute.org; dbSNP rs80338686) (Lek et al. 2016), which is consistent with a recessive carrier frequency. This missense variant meets criteria to be classified as pathogenic for BTD in an autosomal recessive manner based on allelic observations in cases and functional evidence (Pomponio et al. 1997) (ACMG/AMP criteria codes applied: PM3_Strong [upgraded based on multiple occurrences in *trans* with pathogenic variants], PS3).

This individual also harbored a novel splice variant in intron 1 of the *BTD* gene (NM_000060.4:c.44+1G>A, p.?) (ClinVar Variation ID: 370376; https://www.ncbi.nlm.nih.gov/clinvar). This variant, at the time of case assessment, had not been previously reported in patients with BTD and was absent from large population studies. This variant occurs in the invariant region (±1, 2) of the splice consensus sequence and is predicted to abolish the splice site (Alamut Interactive Biosoftware) (see Supplemental Fig. 1A). However, GTEx data support that this exon is alternatively spliced with multiple transcripts across all tissues examined either missing this exon entirely or using alternate 5′ junctions (http://www.gtexportal.org) (see Supplemental Fig. 1B). We also noted that there were no pathogenic variants that had been reported in exon 1. Based on the available data and the uncertainty regarding whether this variant would impact the function of the biologically relevant transcript and resulting biotinidase enzyme, the variant was initially classified as a variant of uncertain significance (VUS) (ACMG/AMP codes applied: PVS1_Supporting, PM2). PVS1 was downgraded to PVS1_Supporting because of the uncertain role of exon 1 in the required function of the gene. Sanger sequencing of parental samples demonstrated that the variants were in *trans*, with the c.1612C>T, p.Arg538Cys variant being inherited from the father and the c.44+1G>A variant being inherited from the mother.

To clarify the significance of the c.44+1G>A variant, the subject's NBS results for biotinidase activity were reviewed. The initial NBS sample sent at ∼24 h of life revealed only 20% enzyme activity (reference levels 30%–100%). A repeat sample generated an in-range value of 32% (see Table 1) (reference levels 30%–100%) leading to no further follow-up based on standard protocols. After the exome sequencing revealed the suspicious *BTD* genotype, to clarify whether the infant might have partial BTD, subsequent diagnostic enzyme testing was performed on a serum sample, which confirmed a low enzyme activity level consistent with a diagnosis of partial BTD (1.5 nmol/min/ml serum) (reference levels 3.5–13.8 nmol/min/ml serum). It should be noted that mean biotinidase activity has been reported at levels of 3.49 ± 0.72 SD nmol/min/ml serum in obligate heterozygotes with profound deficiency alleles (Strovel et al. 2017), suggesting carrier status is insufficient to explain the reduced enzyme level. Based on the newborn's enzyme activity level, the baby was started on biotin supplementation.

**Table 1.** Phenotypic features

Biotinidase deficiency clinical features^a^	Proband
Biotinidase activity level	Low
Other clinical features	Absent

^a^The list of other clinical features are based on the OMIM clinical synopsis (#253260; BTD).

On testing, biotinidase enzyme activity suggested that the c.44+1G>A variant sufficiently impacted function of the biotinidase enzyme to result in partial enzyme deficiency when in *trans* with a variant that is associated with profound deficiency in homozygosity. Incorporation of the biotinidase enzyme testing results enabled application of ACMG/AMP criteria code PS3 elevating the variant to Likely Pathogenic (see Table 2). Subsequently, additional submitted interpretations to ClinVar for this variant (SCV000485680.1) and three other splice variants at the same position (SCV000486767.1, SCV000485974.1, and SCV000485701.1) agree with this conclusion with the first entry citing Stanley et al. (2004), who described that alternative splicing of *BTD* may play a role in regulating its tissue specificity and subcellular localization. However, all of these ClinVar submissions were based on heterozygous variant observations in individuals undergoing carrier screening (P Kang, Counsyl, pers. comm.). Furthermore, it has been reported that one child identified with a novel 107-kb contiguous deletion of three genes in homozygosity, including exon 1 of the *BTD* gene, the entire *HACL1* gene, and the 5′ end of the adjacent *COLQ* gene exon 1, was profoundly affected for BTD (Senanayake et al. 2015). These authors reported that at the time of evaluation, the child's symptoms were apparently solely attributable to the BTD and markedly improved with biotin supplementation (Senanayake et al. 2015). The authors concluded that the deletion of exon 1 in the *BTD* gene results in the inability to synthesize the active enzyme product, as exon 1 contains the initiation site and leader signal sequence of the enzyme, further supporting a critical role for this exon (Senanayake et al. 2015), although a deletion encompassing the entire 5′ region of the gene including exon 1 may have a different impact than a variant that impacts splicing of this exon.

**Table 2.** Genomic findings

Gene	Genomic location	HGVS cDNA	HGVS protein	Parent of origin	Variant interpretation
*BTD*	Chr 3:15686975 (GRCh37)	NM_000060.2: c.1612C>T	p.Arg538Cys	Paternal	Pathogenic
*BTD*	Chr 3:15643402 (GRCh37)	NM_000060.2: c.44+1G>A	p.?	Maternal	Likely pathogenic

Biallelic pathogenic defects in the *BTD* gene lead to BTD presenting with neurocutaneous features, typically diagnosed based on clinical presentation and enzymatic activity. Individuals with partial BTD may develop symptoms upon exposure to metabolic stress and can benefit from biotin supplementation. BTD is highly treatable; but if untreated, young children with profound BTD (<10% biotinidase activity) typically exhibit neurological abnormalities, including seizures, hypotonia, ataxia, developmental delay, vision problems, hearing loss, and cutaneous abnormalities (Wolf 1993). Older children and adolescents with profound BTD frequently exhibit motor limb weakness, spastic paresis, and decreased visual acuity (Wolf 1993). Once features develop, they are usually irreversible, even with vitamin supplementation. Individuals with partial BTD (10%–30% biotinidase activity) may have hypotonia, skin rash, and hair loss, particularly during times of stress (Wolf 1993). Therapy is inexpensive (oral free biotin), with little to no side effects, and may be required lifelong (Wolf 1993). However, it is difficult to anticipate whether this child with borderline enzyme activity levels would develop symptoms if untreated, particularly given that this condition can have variable expressivity even within the same family (Wolf 1993). Because children with partial deficiency may develop symptoms, especially when stressed, and given the potential benefit, and minimal effects, all patients with partial BTD are recommended to be treated with 5 mg of biotin per day as soon as the diagnosis is made (Wolf 1993). Therefore, this infant's treatment regimen of daily vitamin biotin supplementation was instituted primarily as a precaution because of the negligible risks and significant potential benefits associated with this treatment. It should be noted that it was the identification of the *BTD* variants in conjunction with NBS results that led to the diagnosis of partial BTD in the child, resulting in follow-up and reclassification of the variant, which would not normally have been reported as an incidental finding given its initial VUS classification. The combined phenotypic analysis and genotype results were necessary to effectively diagnose and treat the baby—a process now routinely recommended for certain metabolic diseases like BTD (Strovel et al. 2017), but not possible for most other disorders. This case also illustrates the importance of functional testing in helping to decipher whether variants like the novel splice variant are disease causing. This outcome highlights the complexity of interpreting novel splice variants in genes where loss of function is associated with the disease, but for which alternative splicing of multiple transcripts confounds variant interpretation.

## SUMMARY

The *BTD* c.44+1G>A variant, initially classified as a VUS because of occurrence in an alternatively spliced exon, was identified in *trans* with *BTD* p.Arg538Cys, a pathogenic variant for profound BTD. Review of the NBS results identified an initial out-of-range result for BTD, with borderline pass on a rescreen. Confirmation of low enzyme levels upon clinical follow-up in conjunction with genomic sequencing results led to a diagnosis of partial BTD and the baby was started on biotin supplementation. In conclusion, this BabySeq Project case demonstrates the utility of combining genotype and biochemical phenotype information to more effectively guide the interpretation of the novel *BTD* splice site variant (c.44+1G>A variant) as likely pathogenic.

## THE BABYSEQ PROJECT TEAM PARTICIPANTS

Pankaj B. Agrawal,^14^ Alan H. Beggs,^14^ Wendi N. Betting,^15^ Ozge Ceyhan-Birsoy,^16^ Kurt D. Christensen,^15^ Dmitry Dukhovny,^17^ Shawn Fayer,^15^ Leslie A. Frankel,^18^ Casie A. Genetti,^14^ Chet Graham,^16^ Robert C. Green,^15^ Amanda M. Gutierrez,^18^ Maegan Harden,^19^ Ingrid A. Holm,^14^ Joel B. Krier,^15^ Matthew S. Lebo,^16^ Harvey L. Levy,^14^ Xingquan Lu,^18^ Kalotina Machini,^16^ Amy L. McGuire,^18^ Jaclyn B. Murry,^16^ Medha Naik,^18^ Tiffany Nguyen,^15^ Richard B. Parad,^15^ Hayley A. Peoples,^18^ Stacey Pereira,^18^ Devan Petersen,^18^ Uma Ramamurthy,^18^ Vivek Ramanathan,^18^ Heidi L. Rehm,^16^ Amy Roberts,^14^ Jill O. Robinson,^18^ Serguei Roumiantsev,^20^ Talia S. Schwartz,^14^ Tina K. Truong,^14^ Grace E. VanNoy,^14^ Susan E. Waisbren,^14^ Timothy W. Yu^14^

## ADDITIONAL INFORMATION

### Data Deposition and Access

The variant was deposited to ClinVar (https://www.ncbi.nlm.nih.gov/clinvar/) and can be found under accession number SCV000712499.1.

### Ethics Statement

Informed consent was obtained for the proband and family members whose results are reported in this study. The study was approved by the Institutional Review Board of Partners Healthcare and Boston Children's Hospital under protocol #2014P001906 and has received nonsignificant risk status in a pre-Investigational Device Exemption submission to the Food and Drug Administration.

### Acknowledgments

We thank the family for their willingness to participate in the research study.

### Author Contributions

J.B.M. contributed to initial draft of the manuscript; K.M. and O.C.B. contributed to variant interpretation; A.K. and J.B.K. contributed to clinical assessment of the subject and family; M.S.L. contributed to bioinformatics; S.F., C.A.G., and G.E.V. contributed to patient recruitment and clinical disclosure; T.W.Y., P.B.A., and R.B.P. contributed to clinical assessment of the subject and family and clinical disclosure; I.A.H. contributed to clinical assessment of the subject and family and clinical disclosure and supervision; A.L.M. contributed to ELSI assessment of the subject and family; R.C.G. and A.H.B. contributed to supervision; H.L.R. contributed to variant interpretation, supervision and manuscript preparation. BabySeq Investigators contributed to process development, infrastructure deployment, and maintenance. All authors contributed to editing the manuscript and reviewing the final draft.

### Funding

This study was supported by the National Institutes of Health under awards U19HD077671. The content is solely the responsibility of the authors and does not necessarily represent the official views of the National Institutes of Health.

### Competing Interest Statement

The authors have declared no competing interest.

### Referees

Barry Wolf

Anonymous

## Supplementary Material

Supplemental Material

## Footnotes

14 Boston Children's Hospital, Boston, Massachusetts 02115, USA

15 Brigham and Women's Hospital, Boston, Massachusetts 02115, USA

16 Laboratory for Molecular Medicine, Cambridge, Massachusetts 02139, USA

17 Oregon Health and Science University, Oregon 97239, USA

18 Baylor College of Medicine, Houston, Texas 77030, USA

19 Broad Institute, Massachusetts 02141, USA

20 Massachusetts General Hospital, Boston, Massachusetts 02115, USA
