# Supplementary material for: Reconciling newborn screening and a novel splice variant in BTD associated with partial biotinidase deficiency: a BabySeq Project case report
Source: Cold Spring Harb Mol Case Stud. 2018 Aug;4(4):a002873. doi: 10.1101/mcs.a002873 (PMC6071571; doi:10.1101/mcs.a002873)
Supplement: Supplemental Material [file supp_4_4_a002873__index.html]

Reconciling newborn screening and a novel splice variant in BTD associated with partial biotinidase deficiency: a BabySeq Project case report — Supplemental Material 

# Reconciling newborn screening and a novel splice variant in *BTD* associated with partial biotinidase deficiency: a BabySeq Project case report

## Supplemental Material

- Supplemental\_Figure\_1.docx
