# Supplementary material for: Reconciling newborn screening and a novel splice variant in BTD associated with partial biotinidase deficiency: a BabySeq Project case report
Source: Cold Spring Harb Mol Case Stud. 2018 Aug;4(4):a002873. doi: 10.1101/mcs.a002873 (PMC6071571; doi:10.1101/mcs.a002873)

**LEGENDS**

SUPPLEMENTAL FIGURE 1: VARIANT ASSESSMENT. The novel splice variant (c.44+1G>A) was identified in the invariant splice donor region of intron 1. 1A) This splice variant is predicted to result in a disruption of the splicing consensus sequence Alamut (Interactive Biosoftware, Rouen, France). 1B) Exon 1 is indicated with a red box. GTEx data confirms this exon is often spliced out in adult tissues (representative image) ([http://www.gtexportal.org](http://www.gtexportal.org/)). Based on this available data, the variant was initially interpreted as a variant of uncertain significance. This finding prompted review of this subject’s NBS results.

**SUPPLEMENTAL FIGURE 1: VARIANT ASSESSMENT**

1A


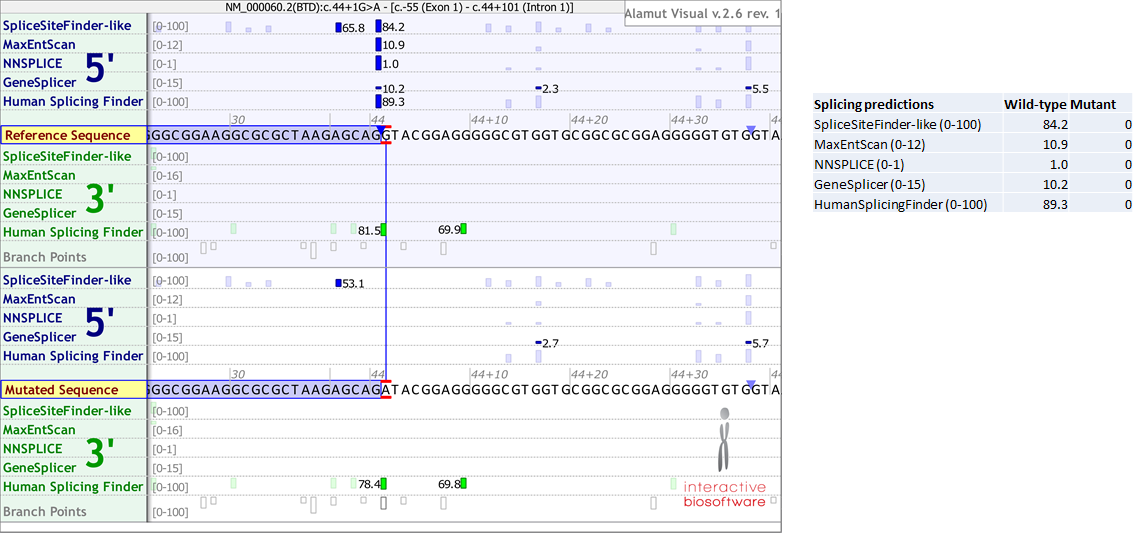


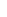


1B.


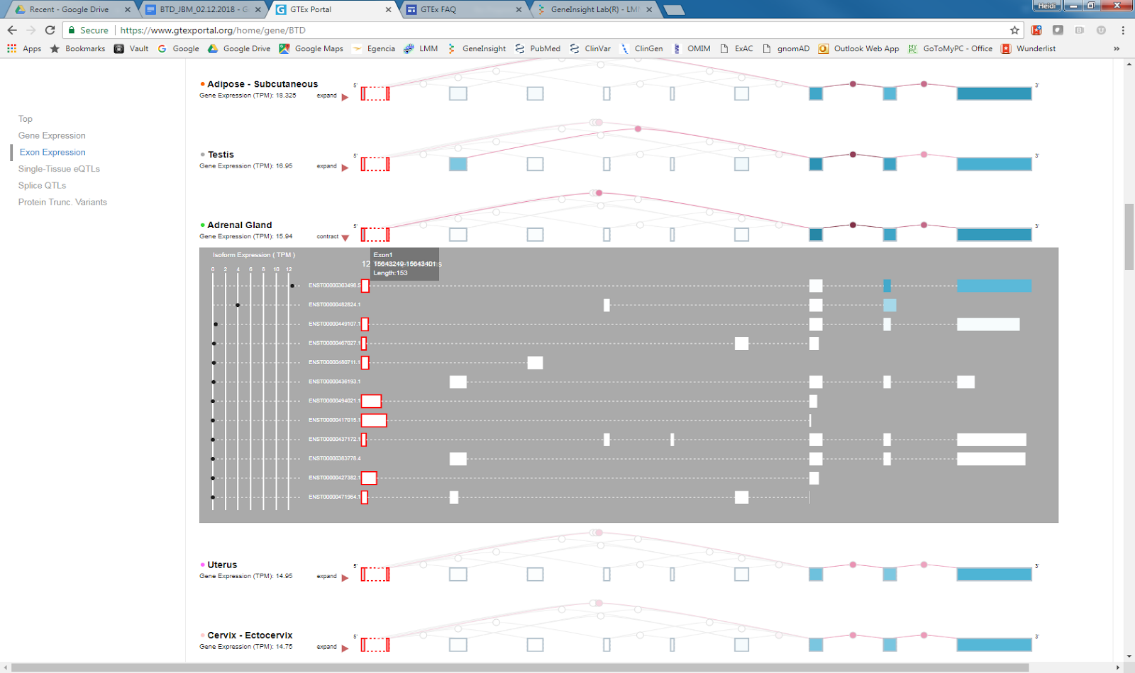

Supplement: Supplemental Material [file supp_mcs.a002873_Supplemental_Figure_1.docx]
